# Case Report: The complete radiological resolution of diffuse cholangitis in a HIV-positive patient with cryptosporidium infection after anti-retroviral therapy

**DOI:** 10.3389/fmed.2026.1686336

**Published:** 2026-04-09

**Authors:** Cheng Bo Li, Shi Tang, Ying Wen

**Affiliations:** The First Affiliated Hospital of China Medical University, Shenyang, China

**Keywords:** anti-retroviral therapy, cholangitis, cryptosporidium, human immunodeficiency virus, immunodeficiency

## Abstract

We present a case of an HIV-positive patient with AIDS cholangiopathy secondary to Cryptosporidium infection. Imaging examination showed intrahepatic and extrahepatic cholangitis without papillary stenosis and extrahepatic bile duct strictures, indicating mild bile duct disease. However, it failed to obtain positive results in fecal microscopy examinations. Alternatively, metagenomic next-generation sequencing (mNGS) of a blood sample identified Cryptosporidium infection. The diagnostic power of mNGS is highly sensitive and can simultaneously identify various pathogens. To avoid irreversible damage to the biliary system, the rapid initiation of anti-HIV therapy restored the function of the immune system and led to the clinical resolution of cryptosporidiosis.

## Background

Among human immunodeficiency virus (HIV)-negative cases, primary sclerosing cholangitis, IgG4-related cholangitis, clonorchiasis, and tumors are the main causes of cholangiopathy. However, cholangiopathy is not uncommon in HIV-infected patients because some cases are underestimated as being in the early stage of this disease without obvious clinical manifestations. The acquired immune deficiency syndrome (AIDS) cholangiopathy is often described as a condition of opportunistic infection-related biliary tract abnormalities, especially in HIV-positive individuals with advanced immunodeficiency (1). *Cryptosporidium*, cytomegalovirus, Microsporidia, Cyclospora, Giardia, and *Mycobacterium avium* intracellulare complex have been reported as the causes of AIDS cholangiopathy (2). Papillary stenosis and extrahepatic duct stricture are severe types of cholangiopathy that require further invasive endoscopic management for biliary obstruction. Here, we report a case of a HIV-positive patient with diffuse cholangitis due to Cryptosporidium infection, who achieved complete recovery within four months after initiating antiretroviral therapy (ART).

## Case presentation

A 17 years and 11 months-old Chinese boy complained of watery diarrhea (4–5 times per day) for six months with a weight loss of 5 kg. The patient did not present with fever or abdominal pain. His abdomen was soft, without tenderness, rebound pain, or hepatosplenomegaly. Then he was confirmed with HIV-1 infection while the HIV screening results of his parents were negative. He reported a history of men who have sex with men (MSM) since the year of 2022. His CD4 + T cell count was 66 cells/μL. His HIV RNA load was not tested at baseline. Leukocyte counts and eosinophils counts in the blood and stool samples were normal. His serum amylase level was 104 U/L (reference range: 35–135 U/L). He had almost normal liver function, including 24 U/L of alanine transaminase (ALT) (reference range: 9–50 U/L), 30 U/L of aspartate aminotransferase (AST) (reference range: 15–40 U/L), 122 U/L of alkaline phosphatase (ALP) (reference range: 45–125 U/L), 61 U/L of gamma-glutamyl transferase (GGT) (reference range: 10–60 U/L), 4.5 μmol/L of the total bilirubin (TBIL) (reference range: 0.0–26.0 μmol/L), and 2.1 μmol/L of direct bilirubin (DBIL) (reference range: 0.0–8.0 μmol/L).

**Figure 1:**
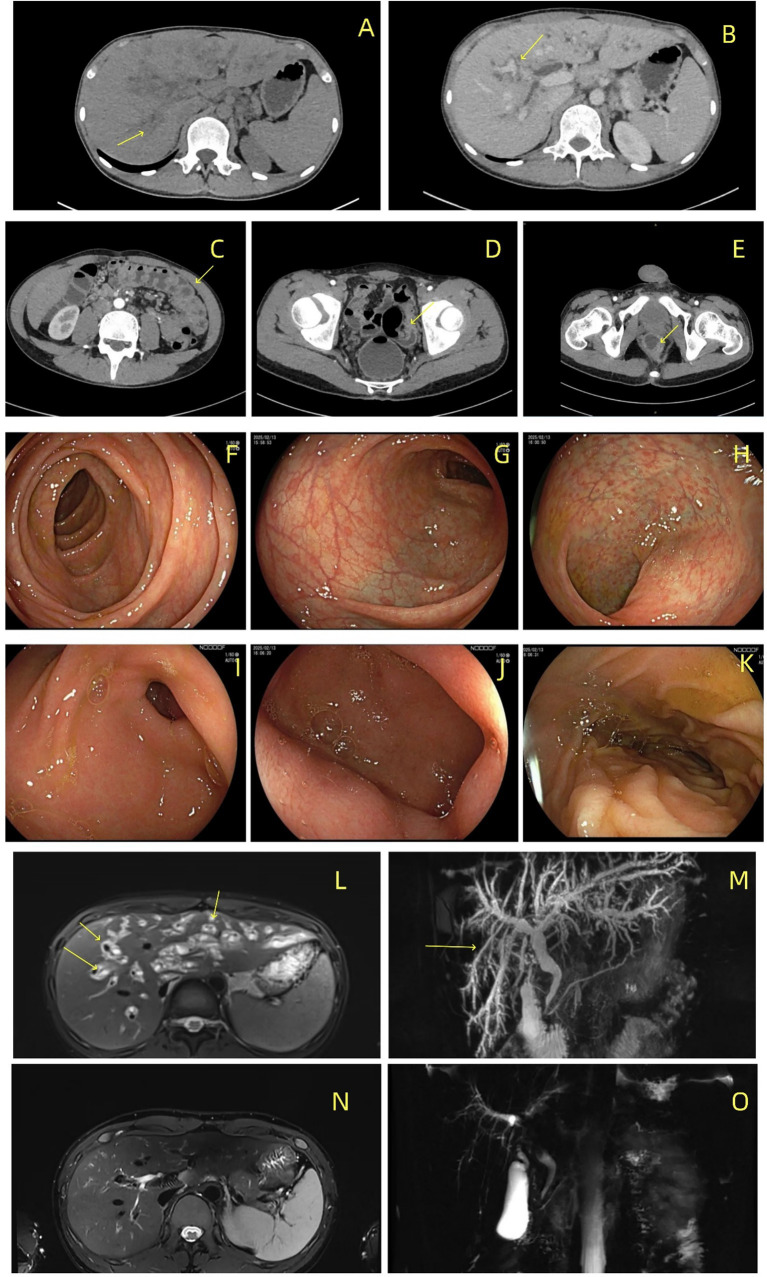
Changes in the bile ducts and the bowel. **(A,B)** Abdominal CT shows dilation of the intrahepatic and extrahepatic bile ducts, with wall thickening and enhancement of the wall (arrows). **(C–E)** Contrast-enhanced abdominal CT demonstrating edematous wall thickening of the transverse, sigmoid, and descending colon. **(F–H)** Colonoscopy revealed no mucosal ulcers or neoplasms (arrows). Only congestion and erosion were observed in the sigmoid colon. **(I–K)** Gastric antrum, duodenal bulb, and duodenal descending part in gastro-duodenoscopy are normal. **(L,M)** Magnetic resonance cholangiopancreatography (MRCP) shows diffuse wall thickening of the intrahepatic and extrahepatic bile ducts, accompanied by mild biliary dilation (arrows). No significant stenosis was observed. **(N,O)** Follow-up MRCP at four months post-antiretroviral therapy initiation demonstrated complete resolution of the biliary abnormalities.

Blood and stool bacterial and fungal cultures were also negative. The parasite was not detected in stool smears (twice). He had a negative T-SPOT result and undetectable serum cytomegalovirus (CMV) DNA. The patient tested negative for *T. gondii* IgM/IgG antibodies, and the serum IgG4 level was normal. Contrast-enhanced CT of the abdomen revealed intra/extrahepatic biliary dilatation and thickened/enhanced biliary (Figures 1A,B) and colonic walls (Figures 1C–E). Magnetic resonance cholangiopancreatography (MRCP) revealed similar findings (Figures 1L,M). Esophagogastroduodenoscopy did not reveal any abnormalities (Figures 1I–K). Colonoscopy revealed mild non-specific spotted congestion and erosion in the sigmoid colon without biopsy samples (Figures 1F–H). A whole blood sample was sent to Shen yang kingmed for clinical laboratory for metagenomic next-generation sequencing (mNGS), and *Cryptosporidium hominis* infection was identified with 70 sequencing reads and 94.96% relative abundance. Then, he initiated ART with Bictegravir Sodium/Emtricitabine/Tenofovir Alafenamide Fumarate. ALP (166 U/L) and GGT (134 U/L) levels were mildly elevated 45 days later. Four months later, an almost normal MRCP manifestation was observed (Figures 1N,O), and the CD4 + T cell count increased to 157 cells/μL. During follow-up, his CD4 + T cell count was 255 cells/μL at six months and 320 cells/μL at nine months. His HIV RNA load was 5,630 copies/mL at one month and 54.7 copies/mL at nine months after ART initiation. The clinical recovery timeline is shown in Table 1.

**Table 1:** The clinical recovery timeline for this case.

Time (months)	0	1	2	4	6	9
CD4 + T (cells/μL) (450–1,590)	66	152	N	157	255	320
HIVRNA (copies/mL) (<20)	N	5,630	N	N	N	54.7
ALT (U/L) (9–50)	24	64	17	9	6	8
AST (U/L) (15–40)	30	38	20	13	15	13
ALP (U/L) (45–125)	122	73	166	54	60	63
GGT (U/L) (10–60)	61	71	134	9	7	7
TBIL (μmol/L) (0–26)	4.5	5.5	3.6	7.6	12.8	9.4
Symptom	Diarrhea	NS	NS	NS	NS	NS

N, not test; NS, no symptom; ALT, alanine transaminase; AST, aspartate aminotransferase; ALP, alkaline phosphatase; GGT, gamma-glutamyl transferase; TBIL, total bilirubin.

## Discussion

The features of this case were the presence of a diffuse thickened biliary wall with mild dilatation and the absence of extrahepatic biliary strictures, which indicated a mild type of bile duct disease. He did not initially present with the common manifestations of AIDS cholangiopathy, such as right upper quadrant pain, cholestasis, and jaundice. Therefore, unlike previous cases (3), further endoscopic retrograde cholangiopancreatography (ERCP) and stent placement/papillary stenosis sphincterotomy were not required in this case. For this case, other causes of cholangiopathy, are gradually excluded. Contrast-enhanced CT of the abdomen and esophagogastroduodenoscopy/colonoscopy examinations did not support tumors. MRCP examination did not support primary sclerosing cholangitis. The serum IgG4 level was normal, which did not support IgG4-related cholangitis. Leukocyte counts and eosinophils counts in the blood were normal, which did not support acute obstructive suppurative cholangitis or clonorchiasis. Importantly, lacking of the target therapy for above causes and the complete radiological resolution of diffuse cholangitis support our analysis for differential diagnosis of cholangiopathy. Clinicians should consider Cryptosporidium as the most common cause of AIDS cholangiopathy. In addition to the ingestion of Cryptosporidium oocysts, sexual transmission is common among MSM (4). Cryptosporidium transmission involves environmental, demographic, and behavioral factors (5). In this case, colonoscopy only revealed mildly non-specific inflammatory lesions in the sigmoid colon, which did not support the typical manifestations of CMV enteritis, intestinal tuberculosis, or amoebic enteritis. As for the lack of colonic biopsy, in the opinion of the endoscopy department physicians in our hospital, they did not routinely obtain biopsy samples for this non-specific Colonoscopy manifestation of this case unless there were some specific requirements beforehand. Therefore, it will be better if we could establish multi-disciplinary team discussion before Colonoscopy examination. Notably, Cryptosporidium mainly colonizes the small intestine, including the duodenum, jejunum, and ileum (6). Therefore, abnormal findings on colonoscopy are often lacking. The intestinal pathology of Cryptosporidium infections is mild. The mechanisms underlying diarrhea include increased intestinal permeability, chloride secretion and malabsorption. Cryptosporidium infections also affect the biliary, pancreatic, and respiratory tracts. Cryptosporidium can be detected in feces, bile, blood, and sputum. The prevalence of Cryptosporidium in HIV-positive individuals is higher than 10% among antiretroviral-naïve and severe immunodeficiency cases with diarrhea (7). Moreover, other immunosuppressed individuals, such as transplantation recipients and primary immunodeficient cases, also have a high risk of disseminated Cryptosporidium infection (8, 9). The immune regulation and effector mechanisms of Cryptosporidium infection have not been completely elucidated (10). Cryptosporidium diagnosis is mainly based on modified Ziehl–Neelsen staining of acid-fast Cryptosporidium oocysts in fecal smears. However, molecular diagnostics are advantageous for identifying Cryptosporidium species and investigating epidemiological data (11). Although the real-time PCR-based method using stool samples is an important diagnostic tool (12), it is not commercially available in our city. Therefore, the application of mNGS using blood or stool samples could be considered an alternative method for the diagnosis of Cryptosporidium infection when positive findings are not obtained by fecal microscopy examination. *Cryptosporidium hominis* and *Cryptosporidium parvum* are the most common Cryptosporidium species that infect humans (13). Although nitazoxanide or paromomycin (unavailable in our region) against Cryptosporidium are generally considered, there is currently no effective treatment for immunodeficient individuals (14). The mainstay of treatment is ART, which ultimately achieves a CD4 + T cell count of >100 cells/μL, clinical resolution, and image normalization. This case highlights that the resolution of lesions after rapid initiation of ART, followed by immune restoration, represents a therapeutic test supporting the diagnosis. This study had some limitations. His diarrhea symptoms improved on admission to our hospital, and he did not provide multiple stool specimens for repeated stool tests during his short hospital stay. This is true that no bile sampling or duodenal aspiration was attempted for tests. The absence of side-viewing endoscopy is another limitation. Although ART alone was opted for this case, which is defensible due to unavailable Nitazoxanide/paromomycin and their less effective in HIV-positive patients, it should be noted that in some patients, adjunct antiparasitic therapy can hasten symptom control. From the patient’s perspective, he understood the hazards of long-term diarrhea and sincerely thanked the doctors for the rapid diagnosis using advanced detection technology and for preventing further severe complications. At this time, he was already 18 years old and provided informed consent for publication. From a privacy safeguards perspective, the doctors firstly provide a direct communication with the patient about a informed consent procedure. It will collect information about the medical history, personal epidemiological information, and radiological images involving abdomen organs, which is based on the purpose of the rapid clinical diagnosis and a reasonable treatment option. Secondly, the patient voluntarily authorizes the doctors to perform the procedure. Then the patient will understand this information adequately. Thirdly, the patient will be involved in the decision making process and be free from coercion. In the end, he signed for agreement with sharing the medical data for public medical research.

## Conclusion

Although no infectious agent has been identified in a subset of cases (15), AIDS cholangiopathy is often thought to be caused by opportunistic infections. According to previously reported three types of presentation (16), this case belongs to the asymptomatic type, not symptomatic biliary obstruction type or pancreatitis type. As a non-invasive examination, MRCP presents an increasing value in diagnosis and follow-up the treatment response of cholangiopathy (17). The combination of imaging examination and available pathogen identification will determine the patterns of cholangiography and guide the subsequent management approaches. Routine screening for Cryptosporidium, particularly in HIV-positive patients with diarrhea, should be recommended. The higher sensitivity of the molecular technique should be widely applied. ART alone also presented a good therapeutic effect in most cases with Cryptosporidium, which was mainly related to an increased CD4 + cell count rather than to the decreased viral load (18). Developing novel treatments and effective vaccines is crucial for controlling this infection.

## Data Availability

The raw data supporting the conclusions of this article will be made available by the authors, without undue reservation.
